# Supplementary material for: Implications of Flagellar Attachment Zone Proteins TcGP72 and TcFLA-1BP in Morphology, Proliferation, and Intracellular Dynamics in Trypanosoma cruzi
Source: Pathogens. 2023 Nov 18;12(11):1367. doi: 10.3390/pathogens12111367 (PMC10675206; doi:10.3390/pathogens12111367)
Supplement: Supplementary file 1 [file pathogens-12-01367-s001.zip › pathogens-2693671-supplementary.pdf]

Supplementary Materials:

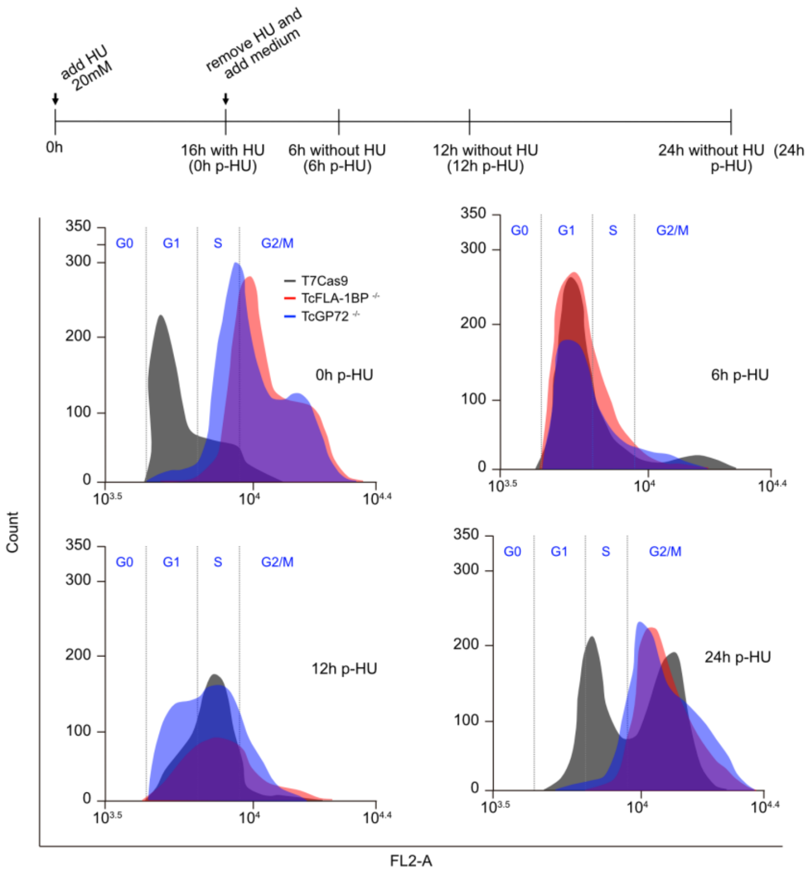

Figure S1. Hydroxyurea (HU) synchronization of epimastigote-form cells of TcGP72 and TcFLA-1BP knockout parasites. Depleted cells were treated with 15 mM HU overnight, washed twice with a growth medium with 10% FSB, and collected at times indicated post-treatment with HU (0, 6, 12, and 24 h p-HU). The collected cells were fixed with ethanol, stained with propidium iodide, and analyzed by flow cytometry. Histograms on the left indicate results from cell sorting for DNA contents (FL2 channel). The data show atypical cellular patterns in knockout parasites.

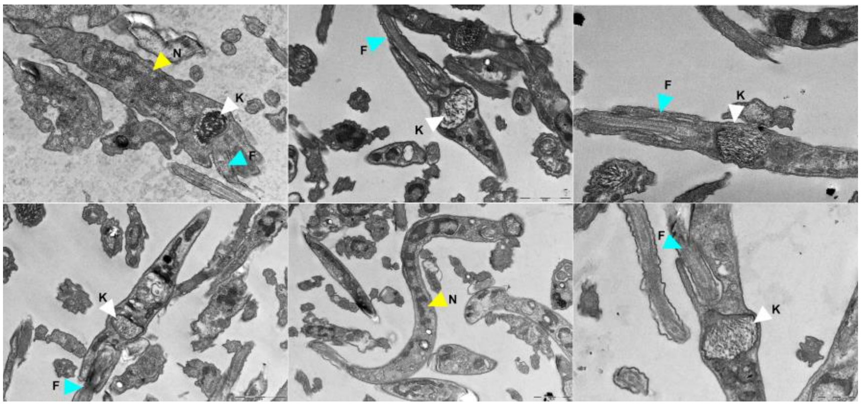

Figure S2. Ultrastructural analysis of metacyclic trypomastigote forms. Ultrastructural analysis of metacyclic trypomastigotes from the TcGP72$^{-/-}$ mutant, purified on a DEAE cellulose column, observed by transmission electron microscopy. The white arrowhead represents the kinetoplast, the

yellow arrowhead the nucleus, and the blue arrowhead the flagellum. The images depict the arrangement of intracellular structures in the parasite depleted for TcGP72.

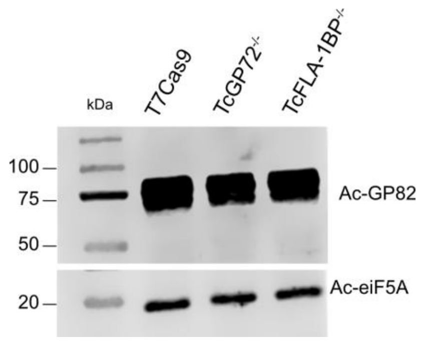

Figure S3. Levels of TcGP82 protein in purified metacyclic trypomastigotes depleted of TcGP72 and TcFLA-1BP. Western blot analysis of total proteins from metacyclic trypomastigotes of Trypanosoma cruzi purified on DEAE cellulose. The numbers in the figure correspond to the positions of molecular weight markers. TcGP82: 82 kDa glycoprotein. TceiF5A: 18 kDa.
